# Probing the Human Brain Functional Connectome with Simultaneous EEG and fMRI

**DOI:** 10.3389/fnins.2016.00302

**Published:** 2016-06-27

**Authors:** David F. Abbott

**Affiliations:** ^1^The Florey Institute of Neuroscience and Mental Health, Austin HospitalMelbourne, VIC, Australia; ^2^The University of MelbourneMelbourne, VIC, Australia

**Keywords:** functional connectivity, brain connectivity, functional connectome, functional magnetic resonance imaging (fMRI), electroencephalography (EEG), simultaneous EEG-fMRI

The human whole-brain “connectome” has been defined as a structural description of the network of elements and connections forming the human brain (Sporns et al., 2005). This can be explored at various spatial scales, from individual neurons to macroscopic neuronal populations. In recent years, usage of the term connectome has broadened beyond structure to include the “functional connectome,” a term coined to describe the collective set of functional connections in the brain (Biswal et al., 2010). The functional connections between brain regions are most often inferred from functional magnetic resonance imaging (fMRI) of the brain at rest. Functional MRI provides a non-invasive means to map brain function at millimeter spatial resolution. However the temporal resolution of fMRI, typically of the order of seconds, limits its ability to capture the full dynamics of neuronal processes. Scalp electroencephalography (EEG) can also be used to construct a functional connectome. Whilst it has limited spatial coverage and much poorer spatial resolution than fMRI, EEG can measure brain activity with the millisecond temporal resolution required to capture neuronal dynamics. It is also possible to acquire EEG and MRI simultaneously, potentially allowing a richer measure of brain connectivity by combining complementary measures. Combining information from EEG and fMRI is not a trivial exercise due to the different sensitivity, temporal and spatial scales of the measures (for a recent review, see Jorge et al., 2014). Each channel of the EEG comprises a superposition of signals arising across a spatial scale of several centimeters, whilst the fMRI signal at each spatial voxel is sampled just once every few seconds. The EEG measure is electrical and therefore directly related to neuronal activity, whereas fMRI relies on a blood oxygenation level dependent (BOLD) contrast that is indirectly related to neuronal activity (Ogawa et al., 1990; Logothetis et al., 2001). Thus, the sensitivity of each modality has different dependencies on underlying physiology and morphometry, and in some cases activity visible on one modality may not be seen on the other (Nunez and Silberstein, 2000). The complementary information that each modality can provide at a whole-brain connectome level has only recently begun to be explored.

Deligianni et al. (2014) have tackled this issue by building upon approaches that have explored simultaneous resting-state fMRI and band-pass filtered EEG signals (e.g., Goldman et al., 2002; see also Jorge et al., 2014) or non-concurrent resting-state fMRI and band-pass filtered MEG signals (e.g., Brookes et al., 2011). Deligianni et al. studied simultaneous EEG and fMRI and sought insight into the underlying signals by determining how well the connectome derived from one modality predicted that derived from the other. Nodes of the connectome were determined from an anatomical parcellation of T1-weighted structural MRI (68 cortical and 14 subcortical regions). Due to the poor spatial resolution of EEG, estimating average time series for each region was necessarily more complex for EEG than the simple voxel averaging approach required for fMRI. The EEG was first filtered into five frequency bands and source localization using beam forming was undertaken for each band. The band-limited power envelope of the EEG was then used to estimate a time series for each cortical gray matter region. For fMRI and for each frequency band of the EEG, a functional connectome was estimated by deriving covariance matrices (effectively a partial correlation analysis). Deligianni et al. then performed statistical inference based on sparse Canonical Correlation Analysis (sCCA) to link EEG and fMRI connectomes, assessing similarity between predicted and estimated connectomes using a measure of geodesic distance between covariance matrices. The authors' detailed description and application of this framework to EEG-fMRI connectivity provides a foundation for its use in future studies.

Deligianni et al. applied the approach to a study of 17 healthy volunteers. Functional connectomes were calculated for about 10.6 min of resting state EEG-fMRI acquired in each individual. Stationarity of functional connectivity was assumed, although this is not a fundamental limitation of the approach; for example the authors note that sliding window correlation could be employed to examine time varying functional connectivity.

The functional connectomes derived by Deligianni et al. from fMRI and EEG exhibit substantial differences. For example the cortical EEG connectomes exhibit a bias toward intra-hemispheric connections, whereas the fMRI connectome tends to exhibit a more uniform mix of inter and intra-hemispheric connections. An interesting observation in the results is that, for cortical regions, prediction performance of fMRI from EEG was relatively stable across EEG frequency bands and better overall than the performance of prediction of EEG from fMRI. This implies there are signatures of resting-state fMRI dynamics across a wide range of EEG frequencies. It also suggests that, at least at the spatial resolution of the atlas-based parcellation used, the band-limited power of the EEG may capture more information on the dynamics of cortical brain activity than fMRI. This is a particularly interesting observation, given that atlas-based parcellation is a common processing strategy for fMRI functional connectivity. One should bear in mind though that this is a relative comparison: neither modality was able to perfectly predict the other, so each modality captures some unique information at this scale. It was also observed that inclusion of subcortical regions resulted in more dissimilar fMRI and EEG connectomes and suggested that fMRI is superior to EEG in capturing dynamical information from those regions.

Whilst simultaneous EEG and fMRI acquisition is now a mature technology mix, EEG quality can potentially be improved further with the addition of motion artifact detection sensors (e.g., Masterton et al., 2007; Abbott et al., 2015). This would be advisable in future studies of functional connectivity with EEG-fMRI, given recent demonstration of spurious correlations driven by in-scanner movement (Fellner et al., 2016). Nevertheless, the greatest potential for future advancement in EEG-fMRI is in methods to make the most of the information captured by each modality. This is highlighted by the work of Deligianni et al., demonstrating with a novel analysis framework the potential to obtain more information on the human functional connectome by utilizing EEG and fMRI together.

## Author contributions

The author confirms being the sole contributor of this work and approved it for publication.

## Funding

DA gratefully acknowledges fellowship funding from the Australian National Imaging Facility. The Florey Institute of Neuroscience and Mental Health acknowledges the strong support from the Victorian Government and in particular the funding from the Operational Infrastructure Support Grant.

### Conflict of interest statement

The author declares that the research was conducted in the absence of any commercial or financial relationships that could be construed as a potential conflict of interest. The reviewer AN and the handling Editor declared their shared affiliation, and the handling Editor states that the process nevertheless met the standards of a fair and objective review.
